# Effects of Maternal Prenatal Multi-Micronutrient Supplementation on Growth and Development until 3 Years of Age

**DOI:** 10.3390/ijerph16152744

**Published:** 2019-08-01

**Authors:** Gang Cheng, Tingting Sha, Xiao Gao, Xialing Wu, Qianling Tian, Fan Yang, Yan Yan

**Affiliations:** Department of Epidemiology and Health Statistics, Xiangya School of Public Health, Central South University, 110 Xiangya Road, Kaifu District, Changsha 410078, Hunan, China

**Keywords:** pregnancy, multi-micronutrient, supplementation, iron, folic acid, growth, development

## Abstract

At present, there is insufficient evidence on whether prenatal multi-micronutrient (MM) supplementation can be an antenatal nutritional intervention or not. This study aimed to explore the sustained effect of prenatal MM supplementation on early childhood health. A total of 939 mother–offspring pairs were followed up in the study between 2015 to 2018 in Changsha, China. Information was mainly collected through household surveys at the ages of 1, 3, 6, 8, 12, 18, 24, and 36 months. General linear models and generalized estimating equation models were used to estimate the effects of maternal prenatal MM compared with IFA supplementation on infant growth and development. Offspring of women who used prenatal MM compared with IFA supplements had lower weight-for-age z score (WAZ) (adjusted β: −0.23, 95% CI: (−0.40, −0.06)) and weight-for-length z score (WLZ) (adjusted β: −0.20, 95% CI: (−0.37, −0.02)) at 3 months old, but a reduced risk of obesity at birth (aRR: 0.30, 95% CI: 0.11–0.78) and being overweight at 3 months old (aRR: 0.52, 95% CI: 0.32–0.84). Moreover, offspring of women who used prenatal MM compared with IFA supplements had significantly higher scores for communication (adjusted β: 0.41, 95% CI: 0.61–0.21), gross motor (adjusted β: 0.68, 95% CI: 0.49–0.88), fine motor (adjusted β: 1.64, 95% CI: 1.45–1.84), problem solving (adjusted β: 0.29, 95% CI: 0.10–0.49), and personal–social (adjusted β: 0.90, 95% CI: 0.70–1.10) skills at 36 months old. Prenatal MM supplementation could result in better infant growth in the first few months of life and improve development scores at the age of 3 years compared with IFA supplementation.

## 1. Introduction

Pregnant women are faced with an increased risk of multiple deficiencies due to the increased requirements of the growing fetus, placenta, and maternal tissues [1]. It is estimated that 41.8% of pregnant women worldwide are anemic, and more than half of these cases are caused by iron deficiency [2]. Micronutrient deficiencies during pregnancy can influence fetal growth and development in utero, subsequent growth and cognitive development in childhood, and health status in adulthood or even when elderly [3]. Daily iron and folic acid supplementation are recommended by the World Health Organization (WHO) for pregnant women to prevent maternal anemia, puerperal sepsis, low birth weight, and preterm birth [4]. WHO and the United Nations Children’s Fund (UNICEF) recommend a daily iodine supplement of 250 µg for pregnant women [5]. Recently, an animal experiment indicated that mild zinc deficiency during pregnancy led to the impairment of spatial learning and memory function in offspring [6]. Maternal vitamin A supplementation enhanced natural antibody concentrations of preadolescent children [7]. Vitamin or mineral supplementation during pregnancy plays an important role in maternal and infant health outcomes.

The effects of prenatal multi-micronutrient (MM) supplementation on fetal and children’s growth and development have also attracted the attention of researchers. Reviews have supported the idea that women who received MM supplementation during pregnancy had fewer low birthweight babies and small-for-gestational-age babies compared with those who received iron–folic acid (IFA) supplementation [1,8]. Antenatal MM supplementation compared with IFA supplementation contributed to a reduction of stillbirths and preterm births [9]. The beneficial effects of maternal MM supplementation on fetal health have been observed consistently [1,8], however, there are some gaps in the evidence regarding the impacts of prenatal MM supplementation on early childhood health. For example, little is known about the effects of prenatal MM supplementation compared with IFA supplementation on the motor and cognitive development of children aged 3 to 6 years old [10]. The mechanisms suggested that fetal undernutrition was linked with abnormal function in postnatal life by persisting changes of hormone secretion and tissue sensitivity [11,12]. Particular attention needs to be paid to children under the age of 3 years, due to the sensitivity and vulnerability of early brain development [13]. If MM supplementation is to be recommended for pregnant women, sustained improvements in early childhood health need to be proven.

Currently, the association of prenatal MM supplementation with early childhood growth has yielded conflicting and inconsistent results [10,14,15,16,17,18]. In a systematic review discussing the health benefits of prenatal MM supplementation in children [10], there were only four studies focusing on the children below the age of 3 years [16,17,18,19]. In the Bangladesh JiVitA trial (*n* = 8529), prenatal MM supplementation was associated with a higher length-for-age z score (LAZ) between 1 and 3 months old, and lower risk of stunting at 1 and 3 months old compared with IFA supplementation [14]. There was the suggestion of lower risk of stunting at 24 months old in a Vietnam study (*n* = 1579) [15]. The weight-for-age z score (WAZ) at 30 months old was increased in the Nepal Janakpur trials (*n* = 917) [16]. A reduction of stunting rate and a higher LAZ during the first 30 months were observed in the Burkina Faso study (*n* = 1294) [17]. However, in a Chinese randomized controlled trial (RCT) following 1388 newborns, the effect of prenatal MM supplementation on child growth was not seen during the first 30 months of life [18]. Taken together, no findings in one study have been replicated in other reports. A Chinese trial (*n* = 1305) showed that prenatal MM supplementation was associated with an increased score of mental development at 1 year old [19], but in the Bangladesh JiVitA trial (*n* = 734) [14], no evidence supported that hypothesis that prenatal MM supplementation had an impact on cognitive and motor function at 2 years old. In sum, very little is known about the effects of prenatal MM supplementation on motor and cognitive development in early childhood, and consequently, the results cannot be replicated [14,19].

At present, studies discussing the effects of prenatal MM supplementation on early childhood growth and development cannot provide strong evidence for antenatal nutritional interventions. Therefore, our cohort study collected prospective data to identify the effects of prenatal MM compared with IFA supplementation on infant linear growth and nutritional status from birth to 3 years old, and motor and cognitive development at 3 years old. This study aimed to test a hypothesis that, compared with IFA, prenatal MM supplementation will lead to sustained improvements in early childhood health.

## 2. Materials and Methods

### 2.1. Study Design and Population

This study was based on an ongoing birth cohort study, which was initiated in 2015 and conducted in three communities in the Kaifu District of Changsha City, Hunan Province, China. A total of 1286 pregnant women gave birth to live births in the three communities from January 2015 to December 2015. These mother–offspring pairs were recruited into the cohort. Additional details of the cohort study are described in previous studies [20,21]. The inclusion criteria for our analysis included: (1) mothers and infants were permanent residents in the Kaifu District; (2) infants had health care records in the Community Health Management Information System (CHMIS); (3) mothers or infants’ caregivers agreed to participate and signed the written informed consents; (4) mothers used MM, IFA, or folic acid (FA) supplements during pregnancy. The exclusion criteria included: (1) mothers had a history of mental illnesses or brain diseases; (2) infants were multiple births or had any congenital diseases.

### 2.2. Data Collection and Procedure

The follow-up surveys were conducted in the regular physical examination of infants at the ages of 1, 3, 6, 8, 12, 18, 24, and 36 months. The mothers or other caregivers of infants were interviewed at home by trained investigators using a self-designed questionnaire. Data on infant anthropometric parameters including length and weight were collected from health care records in the CHMIS and child healthcare handbooks. Information about infant anthropometric parameters, feeding practices, supplement use, and dietary intake were collected at 1, 3, 6, 12, 18, 24, and 36 months, infant development status was collected at 36 months old, and maternal prenatal micronutrient supplementation status and other maternal and infant characteristics were collected when infants were 1 month old.

The sample size was calculated based on the requirements for cohort studies of disease [22]. The estimated incidences of stunting among the MM supplementation group and IFA supplementation group were, respectively, 10% and 20%, according to the previous study [14,15,16,17,18,19]. Assuming that we desired a two-sided test with α = 0.05 and β = 0.15, the total sample size needed for the study was 448.

### 2.3. Measurement of Prenatal Micronutrient Supplementation Status

Prenatal micronutrient supplementation status was assessed by two questions through a face-to-face interview. (1) Have you ever used FA supplements during pregnancy (yes or no); (2) What kinds of nutritional supplements have you used during pregnancy (none, iron supplements, multivitamin supplements, zinc supplements, calcium supplements, maternal formula milk, or others). Prenatal micronutrient supplementation status was classified into three groups: MM supplementation, IFA supplementation, and FA supplementation. The MM supplementation group was defined as the mothers who took iron supplements, FA supplements, multivitamin supplements, zinc supplements, and maternal formula milk together. The IFA supplementation group was defined as the mothers who used both iron and FA supplements. The FA supplementation group was defined as the mothers who only used FA supplements.

### 2.4. Measurement of Infant Growth and Development

The lengths and weights of the infants were measured at 1, 3, 6, 12, 18, 24, and 36 months old by trained nurses using standardized methods. Infant length was measured using a leather measuring tape that was precise to 0.1 cm. Infant weight was tested twice on an electronic scale to the nearest 0.1 kg, excluding the weight of clothes, shoes, and caps. Length and weight were then converted into length-for-age z score (LAZ), weight-for-age z score (WAZ), and weight-for-length z score (WLZ) according to 2006 World Health Organization (WHO) child growth standards [23]. ‘Stunting,’ ‘underweight,’ and ‘wasting’ were defined as a LAZ, WAZ, and WLZ of <−2, respectively. ‘Overweight’ and ‘obese’ were defined as a WAZ and WLZ of >2, respectively.

Infant development status was assessed at 36 months old with the use of Ages and Stages Questionnaires, Third Edition (ASQ-3) [24]. The ASQ-3 is a set of age-specific developmental screening questionnaires to be completed by parents [24]. In our study, the ASQ-3 was translated into Chinese and was culturally appropriate for Chinese children [25,26]. Its reliability and validity have been evaluated and shown to be satisfactory [25,26]. It concerns five developmental domains: communication, gross motor, fine motor, problem-solving, and personal–social skills. Each domain contains six questions about important developmental milestones appropriate for the specific age. Response options for each question are indicated as “yes” (10 points) when the behavior is present, “sometimes” (5 points) when the behavior is emerging, or “not yet” (0 points) when the behavior is absent. Raw scores are calculated for each developmental domain by summing the scores of the six questions. When one domain scores 1 SD below the normative mean, the domain was regarded as a delay in our study. When one or more domain scores 1 SD below the normative mean, the total development was regarded as a delay in our study.

### 2.5. Measurement of Covariates

Potential confounding variables were identified from previous literature [14,19,20]. Socio-demographic factors included maternal age (<25, 25−29, 30–34, or ≥35 years old), maternal education (junior school or below, senior high school, or college or above), average monthly household income (≤2000, 2001–5000, or >5000 RMB), and infant gender (male or female). Maternal characteristics included postpartum BMI (<18.5, 18.5–23.9, 24–27.9, or ≥28 kg/m^2^), height (m), parity (1, 2, or ≥3), calcium supplementation (yes or no), gestational dietary intake (≥3 times a week or <3 times a week), passive smoking during pregnancy (yes or no), and any alcohol use during pregnancy (yes or no). Based on the recommendations from the Working Group on Obesity in China [27], BMI was categorized into four levels: <18.5 kg/m^2^ (underweight), 18.5–23.9 kg/m^2^ (normal weight), 24–27.9 kg/m^2^ (overweight), and 28 kg/m^2^ (obese). Gestational dietary intake was measured by the frequency of eating meat or liver, fish or seafood, egg, milk products, and fruits or vegetables [9,14]. Passive smoking means women were exposed to secondhand smoke for over 15 min a day [21]. Alcohol use during pregnancy was measured by asking mothers “Did you drink any alcohol during your last pregnancy?” [28]. Infant characteristics included birthweight (<2500, 2500–3999, or ≥4000 g), length at birth (cm), gestational age (<37, 37–41, or ≥42 weeks), feeding practices, supplement use (yes or no), and dietary intake (≥3 times a week or <3 times a week). Infant feeding practices included whether infants were breastfed (yes or no) and provided with formula (yes or no). Infant supplement use included calcium, vitamin D, iron, and zinc supplementation. Infant dietary intake was measured by the frequency of eating meat or liver, fish or seafood, egg, and fruits or vegetables [9,14].

### 2.6. Statistical Analysis

Continuous variables were described as means ± standard deviation (SDs) if normally distributed and as median (interquartile range (IQR)) if not. Categorical variables were described as number (percent). The differences in maternal and infant characteristics between three groups of prenatal micronutrient supplementation status were compared using the chi-square test for continuous variables and one-way ANOVA for categorical variables.

General linear models were used to estimate the effects of maternal prenatal MM compared with IFA supplementation on infant linear growth and development score at different age points. We specified the distribution as “normal” and the link function as “identity.” Estimates of the regression coefficients (β) and corresponding 95% confidence intervals (CIs) quantified the extent of effects. Model 1 was adjusted for socio-demographic factors, including maternal age, maternal education, average monthly household income, and infant gender. Model 2 was adjusted for all confounding factors, including maternal age, maternal education, average monthly household income, infant gender, maternal postpartum BMI, maternal height, maternal calcium supplementation, gestational dietary intake, passive smoking during pregnancy, any alcohol use during pregnancy, maternal parity, infant birthweight, length at birth, gestational age, infant feeding practices, infant supplement use, and infant dietary intake. At the age of birth, infant birth weight and length at birth were not adjusted. General linear models were also used to estimate the effects of maternal prenatal MM compared with IFA supplementation on infant nutritional status and development delay at different age points. Here, the distribution was specified as “binomial” and the link function as “log.” Relative risk (RR) and corresponding 95% CI quantified the extent of effects. The adjusted analyses also included Model 1 and Model 2.

Generalized estimating equation (GEE) models were used to estimate the effects of maternal prenatal MM compared with IFA supplementation on infant linear growth within different age intervals. We specified the covariance matrix as “robust estimator,” the working correlation matrix as “unstructured,” the distribution as “normal,” and the link function as “identity.” The adjusted analyses also included Model 1 and Model 2. GEE models were also used to estimate the effects of maternal prenatal MM compared with IFA supplementation on infant nutritional status within different age intervals. Here, the distribution was also specified as “binomial” and the link function as “log.” The adjusted analyses also included Model 1 and Model 2.

Missing values were estimated by multiple imputations, which is increasingly recommended to adjust for the bias and loss of information [29,30]. A two-tailed *p*-value < 0.05 was regarded as statistically significant. All the statistical analyses were performed using the software IBM SPSS Statistics, version 22.0 (IBM, New York, NY, USA).

### 2.7. Ethical Considerations

The cohort study was approved by the Independent Ethics Committee of Clinical Pharmacology Institute, Central South University, Changsha, China (Ethical code: CTXY-130041-3-2).

## 3. Results

**Figure 1:**
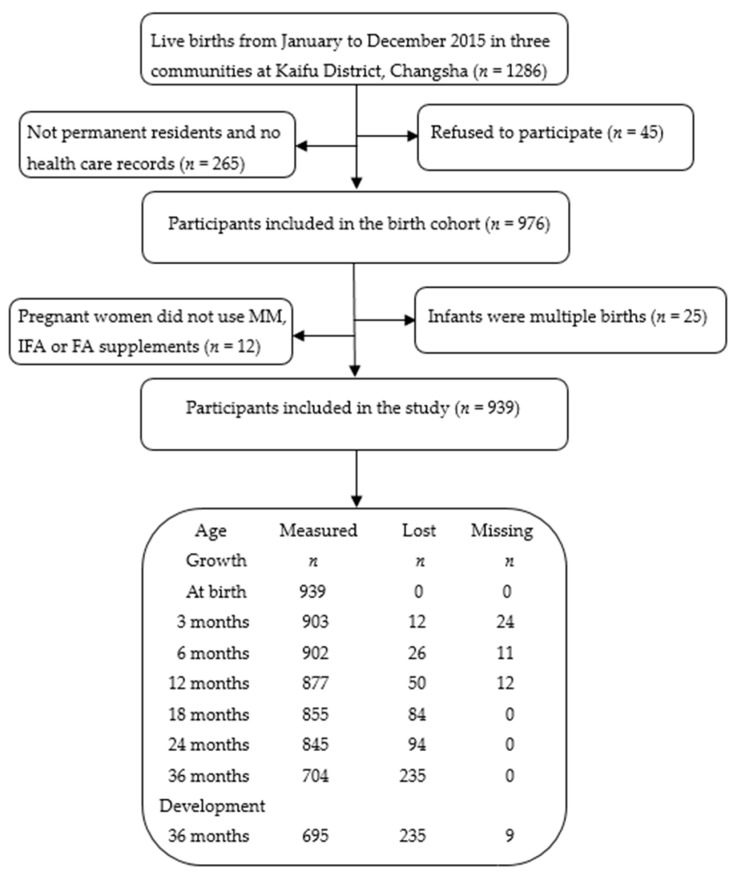
Participant flow diagram for effects of maternal prenatal multi-micronutrient supplementation on growth and development until 3 years of age. (Abbreviation: MM, multi-micronutrient; IFA, iron–folic acid; FA, folic acid).

A participant flow diagram of this cohort study is shown in Figure 1. After excluding the participants who were not permanent residents and did not have health care records (*n* = 265), refused to participate (*n* = 45), did not use MM, IFA, or FA supplements (*n* = 12) during pregnancy, and gave birth to multiple births (*n* = 25), 939 mother–offspring pairs were included in our study. In addition, there were 12(1.3%), 26(2.8%), 50(5.3%), 84(8.9%), 94(10.0%), and 235(25.0%) infants who were lost to follow-up at 3 months, 6 months, 12 months, 18 months, 24 months, and 36 months, respectively.

Table 1 shows the maternal and child characteristics in the different prenatal micronutrient supplementation groups. The rates of prenatal MM supplementation, IFA supplementation, and FA supplementation were 27.1% (254/939), 36.5% (343/939), and 36.4% (342/939), respectively. Women who had higher education (*p* = 0.013) and were primiparous (*p* = 0.014) were more likely to have taken MM supplements. The population with missing data and those estimated by multiple imputations had similar maternal and child characteristics in the different prenatal supplementation groups (see Supplemental Table S1). The mean LAZ, WLZ, and WAZ and prevalence of being underweight, stunting, wasting, being overweight, and obesity by infant age in the cohort are provided in Supplemental Tables S2 and S3.

Table 2 shows the effects of maternal prenatal MM compared with IFA supplementation on infant linear growth and development score at different age points. In both unadjusted and adjusted analyses, offspring in the MM group had significantly lower WAZ (adjusted β: −0.23, 95% CI: (−0.40, −0.06)) and WLZ (adjusted β: −0.20, 95% CI: (−0.37, −0.02)) at 3 months old than those in the IFA group. Moreover, offspring in the MM group had significantly higher scores of communication (adjusted β: 0.41, 95% CI: 0.61–0.21), gross motor (adjusted β: 0.68, 95% CI: 0.49–0.88), fine motor (adjusted β: 1.64, 95% CI: 1.45–1.84), problem solving (adjusted β: 0.29, 95% CI: 0.10–0.49), and personal–social (adjusted β: 0.90, 95% CI: 0.70–1.10) skills than those in the IFA group at 36 months old.

Table 3 shows the effects of maternal prenatal MM compared with IFA supplementation on infant linear growth within different age intervals. In both unadjusted and adjusted analyses, we did not observe that LAZ, WAZ, and WLZ were significantly different between MM and IFA group within any age intervals.

Table 4 shows the effects of maternal prenatal MM compared with IFA supplementation on nutritional status at different age points. In both unadjusted and adjusted analyses, infants born to MM compared with IFA-supplemented mothers exhibited a lower risk of obesity at birth (aRR: 0.30, 95% CI: 0.11–0.78) and being overweight at 3 months old (aRR: 0.52, 95% CI: 0.32–0.84). However, we did not observe that the risks of being underweight, stunting, and wasting were significantly different between the MM and IFA groups at any age points.

Table 5 shows the effects of maternal prenatal MM compared with IFA supplementation on nutritional status within different age intervals. In the adjusted analysis only, infants born to MM- compared with IFA-supplemented mothers exhibited a lower risk of being overweight from 3 to 12 months old (aRR: 0.62, 95% CI: 0.43–0.89). However, we did not observe that the risks of being underweight, stunting, wasting, and obesity were significantly different between MM and IFA group within any age intervals.

Table 6 shows the effects of maternal prenatal MM compared with IFA supplementation on development delay. In both unadjusted and adjusted analyses, we did not observe that the risks of the delay in any developmental domain at 36 months old were significantly different between the MM and IFA groups.

## 4. Discussion

In this study, we found that offspring of women who used prenatal MM supplements had lower WAZ and WLZ at 3 months old and a reduced risk of obesity at birth and being overweight at 3 months old compared with IFA supplements. In addition, the offspring of women who used prenatal MM supplements compared with IFA supplements had increased scores in communication, gross motor, fine motor, problem-solving, and personal–social skills at 36 months old. After adjusting for socio-demographic factors or all confounding variables, the results were the same as the unadjusted.

The recent maternal prenatal micronutrient supplementation status in the urban area of China can be seen from our birth cohort. One third of pregnant women were willing to use FA supplements, but ignored the iron supplements; they could not reach the recommendation from WHO for pregnant nutrition [4]. A cross-sectional study found that only 19.8% of women reported taking iron supplements during pregnancy in northwest China from 2010 to 2013 [31]. The rate may have been much lower because part of this group of women came from rural areas. Obviously, most pregnant women from China failed to realize the importance of iron supplements. Relevant government agencies need to pay attention to this problem and propose ways to solve it. In our cohort, the number of pregnant women who took FA supplements was almost the same as who took IFA supplements. Pregnant women should use MM or IFA supplements, not only one single micronutrient, considering their increased nutritional requirements. In our study, women who had higher education and less parity were more likely to have taken MM or IFA supplements, so interventions should be targeted at multiparous mothers and those with lower education.

Previous studies have reported the effects of prenatal MM compared with IFA supplementation on infant linear growth. In the Bangladesh JiVitA trial, a lower predicted WAZ at 6, 12, 24 months old and WLZ at 1, 3, 6, 12, and 24 months old were seen in the MM group than in the IFA group after adjusting for size at birth [14]. In our study, the growth velocity during postpartum life also became slower in the MM group compared with the IFA group. The Burkina Faso study showed that MM supplementation resulted in a higher LAZ during the first 30 months and WLZ at 30 months old [17]. However, all of these results were not observed in our study. Women in this trial took the MM supplementation based on United Nations International Multiple Micronutrient Preparation (UNIMMAP) and recommended for trial purposes by UNICEF/United Nations University/World Health Organization, which contains one recommended dietary allowance of 15 micronutrients [32]. A more variety and more precise dose were required in the trial than those in our study, which may be the cause of the difference. The Vietnam study showed that higher mean birthweight and taller height at 2 years of age were recorded in the districts receiving MM supplements than in those receiving IFA supplements [15]. Our study did not show these findings. The composition of MM supplementation in this trial was also based on UNIMMAP. The Nepal Janakpur trials found that mean weight at birth and at 2.5 years old were higher in the MM group than those in the IFA group [16]. In general, mean WAZ converted by weight will be smaller than mean weight. It then becomes harder to test the statistical difference of mean WAZ than that of mean weight between two groups. The Chinese RCT showed that no significant differences were observed in LAZ, WAZ, and WLZ during the first 30 months among MM, IFA, and FA groups [18]. The results were basically consistent with ours.

Several studies have reported the effects of prenatal MM compared with IFA supplementation on infant nutritional status. The JiVitA-3 trial found that MM compared with IFA supplementation was associated with a lower risk of stunting and being underweight at 3 months old [14]. The difference was not observed in our study. A possible explanation could be due to the different prevalence of stunting and being underweight in the two study areas. The prevalence of stunting and being underweight at 3 months old in rural Bangladesh was 24.1% and 25.0%, respectively. In our cohort, the prevalence of stunting and being underweight at 3 months old was 1.6% and 0.9%, respectively, much lower than those in rural Bangladesh. Our study focused on an urban population, which may cause lots of differences in results. The Burkina Faso study showed that MM supplementation resulted in a reduction of the stunting rate compared with IFA supplementation during the first 30 months [17]. The multivariate analysis in this study was only adjusted for parity and gestational age at delivery. After adjusting for the size at birth, the differences might be not seen at the later ages of 6, 12, and 24 months [14]. The Vietnam study suggested that the stunting rate at 24 months old in the district receiving MM was significantly less than that in the district receiving IFA [15]. This study only used the chi-square test to compare the stunting rate between the MM and IFA groups, not adjusting for any confounding factors, so there was some uncertainty on their results.

A few studies have reported the effects of prenatal MM compared with IFA supplementation on infant development. The JiVitA-3 trial did not observe any difference in composite scores of infant cognitive, language, and motor performance between the MM and IFA groups at 24 months old [14]. One possible explanation in this case is that the Bayley-III test, as the only screening tool, was not sufficiently sensitive to discern abnormal cognitive and motor development in early childhood [14]. The Chinese RCT found that MM supplementation compared with IFA supplementation was associated with an increased mean raw score of mental development at 12 months old [19]. Similar results were found in our study. Moreover, a randomized trial found that maternal MM supplementation had long-term benefits for child cognitive development at the age of 9–12 years [33]. In our study, the development scores were significantly different between the MM group and IFA group, but a difference of development delay was not observed between the two groups.

There was evidence that maternal prenatal MM supplementation increased birth weight [15,16,34,35,36], reduced the prevalence of small for gestational age births [35], and reduced early infant mortality rates [34,35]. The positive effects persisted into early childhood, with increases in both weight and body size in the first few years of life [15,16]. In contrast, the studies in Bangladesh and China failed to reveal positive effects during the first 24 and 30 months [14,18]. Prenatal MM supplementation may be associated with slower growth velocity during the postpartum period compared with IFA supplementation. This association did not increase the rates of being underweight, stunting, or wasting, but reduced the rate of obesity and being overweight; it was essentially good for infant growth. One possible mechanism of the effects was that catch-up growth occurred in those infants born with low birthweight or small for gestational age. Prenatal MM supplementation resulted in increased birth weight and better birth outcomes than IFA supplementation [15,16,34,35,36]. The number of infants born with low birthweight or small for gestational age was higher in the IFA group. They showed faster post-natal growth and gained weight and height more rapidly as a result of recovery from undernutrition in-utero [37]. The phenomenon was assumed to be “catch-up” growth [37]. Another possible mechanism was that the environmental factors played a more dominant role in postnatal linear growth. We guessed that the newborns in IFA groups exhibited poor growth, then their parents gave them more food and richer nutrition. Protein and macronutrients during early childhood are of great importance in better growth achievements [38].

Prenatal MM supplementation is hypothetically preferable to supplementation with FA plus iron for benefits of infant development [19]. Apart from FA and iron, there is evidence that other micronutrients play a role in children’s nervous development. While severe zinc deficiency during pregnancy can lead to overt fetal brain malformations, gestational suboptimal zinc nutrition can have long-term effects on the nervous systems of offspring [39]. The evidence unequivocally supports that severe iodine deficiency in pregnancy leads to the impairment of brain development in the child [40]. Two intervention trials found that children of moderately iodine-deficient mothers supplemented earlier rather than later in pregnancy had improved neurodevelopment [40]. Multiple vitamins administered together were found to result in a significantly reduced risk for developmental delay on the motor scale among infants who were born to HIV-positive mothers [41]. Preclinical studies demonstrated that zinc was essential for normal neurogenesis, myelination, synaptogenesis, neuronal apoptosis, and regulation of neurotransmitter release in the nervous system [39,42], and iodine was essential for synaptic plasticity, neuronal migration, and glutamatergic signaling [43,44]. Animal experiments showed that maternal vitamin B6 deficiency altered the development of the dopaminergic system [45]. Dopamine has an important role in the procedural memory system, underlying the processes of perceptual, motor, and cognitive skills [33].

There are several strengths to our study. The age of the children observed in this study was extended to 3 years, including all ages in the previous studies, so as to review and summarize all previous studies. The present study has provided new evidence for the effects of prenatal MM supplementation compared with IFA supplementation on the motor and cognitive development of children aged 3 years old, which had previously been paid relatively little attention. Our study also has several limitations. First, the dosage, composition, and frequency of MM supplementation in the cohort were not clear. The MM supplementation in most of the trial studies was in accordance with the recommendation of UNIMMAP. Therefore, some results of this study may be slightly different from those of trial studies. Second, our study only considered the nutritional supplements, and did not add the intake from diet. However, women’s high micronutrient dietary quality before conception and throughout pregnancy had no overall effect on birth weight [46]. Third, recall bias might exist because we collected the information about prenatal micronutrient supplementation status at 1 month after giving birth. Fourth, detection bias might exist because most information came from interviewing mothers or other caregivers, and was not verified again. Fifth, the sample size in our cohort might be not large enough to reveal the true effects of prenatal MM supplementation on infant growth, which achieved statistical significance in some studies.

## 5. Conclusions

This study suggests that prenatal MM supplementation could result in better infant growth in the first few months of life and improve development scores at the age of 3 years, compared with IFA supplementation. A prenatal intervention of MM supplementation contributed to medium-term benefits for postpartum growth in infancy and development in early childhood. In the future, larger RCTs are warranted to confirm the effects of prenatal MM supplementation on the motor and cognitive development of children aged 3 to 6 years old.

## Figures and Tables

**Table 1:** Maternal and child characteristics according to prenatal micronutrient supplementation status (*n* = 939).

Characteristics	MM (*n* = 254)	IFA (*n* = 343)	FA (*n* = 342)	*p*
Maternal characteristics				
Age (years)				0.618
<25	10(3.9)	14(4.1)	23(6.7)	
25–29	128(50.4)	159(46.4)	159(46.5)	
30–34	86(33.9)	123(35.9)	117(34.2)	
≥35	30(11.8)	47(13.7)	43(12.6)	
Education				0.013 *
Junior school or below	6(2.4)	12(3.5)	15(4.4)	
Senior high school	23(9.1)	38(11.1)	59(17.3)	
College or above	225(88.6)	293(85.4)	268(78.4)	
Average monthly household income (RMB)				0.079
≤ 2000	13(5.1)	10(2.9)	13(3.8)	
2001–5000	134(52.8)	165(48.1)	195(57.0)	
> 5000	107(42.1)	168(49.0)	134(39.2)	
Postpartum BMI (kg/m^2^)				0.642
<18.5	6(2.4)	7(2.0)	6(1.8)	
18.5–23.9	144(56.7)	207(60.3)	188(55.0)	
24–27.9	82(32.3)	106(30.9)	112(32.7)	
≥28	22(8.7)	23(6.7)	36(10.5)	
Height (m)	1.60 ± 0.08	1.60 ± 0.07	1.60 ± 0.04	0.687
Parity				0.014 *
Primiparous	194(76.4)	224(65.3)	238(69.6)	
Multiparous	60(23.6)	119(34.7)	104(30.4)	
Infant characteristics				
Gender				0.259
Male	142(55.9)	117(51.6)	168(49.1)	
Female	112(44.1)	166(48.4)	174(50.9)	
Birthweight (g)				0.775
<2500	3(1.2)	5(1.5)	8(2.3)	
2500–3999	232(91.3)	317(92.4)	311(90.9)	
≥4000	19(7.5)	21(6.1)	23(6.7)	
Length at birth (cm)	50.01 ± 1.00	49.98 ± 0.77	49.89 ± 0.92	0.194
Gestational age (weeks)				0.732
<37	9(3.2)	11(3.1)	13(3.8)	
37–41	243(96.3)	325(96.3)	325(95.3)	
≥42	2(0.5)	7(0.6)	4(0.9)	

Abbreviation: MM, multi-micronutrient; IFA, iron–folic acid; FA, folic acid. Values are *n* (%) or means ± SDs. One-way ANOVA was used to compare means; the chi-square test was used to compare proportions. Level of significance: * *p* < 0.05.

**Table 2:** Effects of maternal prenatal multi-micronutrient supplementation compared with iron–folic acid supplementation on infant linear growth and development score at different age points.

Outcomes	Unadjusted Analysis		Model 1 ^a^		Model 2 ^b^	
	β	95% CI	β	95% CI	β	95% CI
LAZ						
At birth	0.01	(−0.15, 0.17)	0.03	(−0.13, 0.19)	0.04	(−0.13, 0.21)
3 months	−0.13	(−0.29, 0.04)	−0.12	(−0.28, 0.05)	−0.11	(−0.28, 0.06)
6 months	−0.07	(−0.23, 0.09)	−0.07	(−0.23, 0.10)	−0.10	(−0.27, 0.08)
12 months	−0.05	(−0.22, 0.12)	−0.04	(−0.21, 0.13)	−0.04	(−0.22, 0.14)
18 months	−0.02	(−0.19, 0.15)	−0.02	(−0.20, 0.15)	−0.02	(−0.21, 0.16)
24 months	0.05	(−0.12, 0.23)	0.03	(−0.15, 0.20)	0.03	(−0.16, 0.21)
36 months	0.00	(−0.16, 0.17)	−0.01	(−0.18, 0.15)	−0.01	(−0.20, 0.19)
WAZ						
At birth	0.03	(−0.13, 0.19)	0.02	(−0.15, 0.18)	0.14	(−0.04, 0.32)
3 months	−0.24	(−0.40, −0.08) **	−0.22	(−0.38, −0.05) **	−0.23	(−0.40, −0.06) **
6 months	0.08	(−0.17, 0.16)	0.00	(−0.16, 0.17)	−0.03	(−0.20, 0.14)
12 months	0.06	(−1.11, 0.22)	0.07	(−0.10, 0.24)	0.05	(−0.13, 0.22)
18 months	−0.07	(−0.24, 0.10)	−0.07	(−0.25, 0.10)	−0.11	(−0.29, 0.08)
24 months	0.06	(−0.11, 0.23)	0.04	(−0.13, 0.22)	0.01	(−0.17, 0.19)
36 months	0.05	(−0.13, 0.22)	0.04	(−0.14, 0.22)	−0.01	(−0.20, 0.19)
WLZ						
At birth	−0.03	(−0.19, 0.13)	−0.02	(−0.18, 0.14)	0.10	(−0.08, 0.28)
3 months	−0.20	(−0.36, −0.03) *	−0.18	(−0.35, −0.02) *	−0.20	(−0.37, −0.02) *
6 months	0.05	(−0.11, 0.21)	0.06	(−0.11, 0.22)	0.04	(−0.13, 0.21)
12 months	0.10	(−0.07, 0.27)	0.11	(−0.06, 0.28)	0.08	(−0.10, 0.25)
18 months	−0.07	(−0.24, 0.10)	−0.07	(−0.24, 0.10)	−0.11	(−0.39, 0.17)
24 months	0.03	(−0.15, 0.20)	0.02	(−0.16, 0.19)	−0.02	(−0.20, 0.16)
36 months	0.07	(−0.14, 0.27)	0.07	(−0.13, 0.27)	−0.01	(−0.20, 0.19)
Development score at 36 months						
Communication	0.42	(0.61, 0.23) ***	0.35	(0.54, 0.16) ***	0.41	(0.61, 0.21) ***
Gross motor	0.56	(0.39, 0.77) ***	0.64	(0.46, 0.83) ***	0.68	(0.49, 0.88) ***
Fine motor	1.79	(1.60, 1.98) ***	1.80	(1.61, 1.99) ***	1.64	(1.45, 1.84) ***
Problem solving	0.23	(0.04, 0.42) *	0.23	(0.04, 0.42) *	0.29	(0.10, 0.49) **
Personal social	0.74	(0.55, 0.93) ***	0.92	(0.73, 1.11) ***	0.90	(0.70, 1.10) ***

Abbreviation: β, regression coefficient; CI, confidence interval; LAZ, length for age Z scores; WAZ, weight for age Z scores; WLZ, weight for length Z scores. Level of significance: * *p* < 0.05, ** *p* < 0.01, *** *p* < 0.001. General linear models were used to assess the effects. ^a^ Model 1 included the variables of maternal age, maternal education, average monthly household income, and infant gender. ^b^ Model 2 included the variables of Model 1 plus maternal postpartum BMI, maternal height, maternal calcium supplementation, gestational dietary intake, passive smoking during pregnancy, alcohol use during pregnancy, maternal parity, infant birthweight, length at birth, gestational age, infant feeding practices, infant supplement use, and infant dietary intake. At the age of birth, infant birthweight and length at birth were not adjusted.

**Table 3:** Effects of maternal prenatal multi-micronutrient supplementation compared with iron–folic acid supplementation on infant linear growth within different age intervals.

Outcomes	Unadjusted Analysis		Model 1 ^a^		Model 2 ^b^	
	β	95% CI	β	95% CI	β	95% CI
LAZ						
3 to 12 months	−0.08	(−0.22, 0.06)	−0.08	(−0.21, 0.06)	−0.08	(−0.21, 0.06)
3 to 24 months	0.00	(−0.10, 0.10)	−0.01	(−0.10, 0.09)	−0.03	(−0.12, 0.07)
3 to 36 months	0.00	(−0.09, 0.09)	−0.01	(−0.10, 0.08)	−0.03	(−0.12, 0.06)
WAZ						
3 to 12 months	−0.07	(−0.21, 0.07)	−0.06	(−0.20, 0.07)	−0.09	(−0.22, 0.04)
3 to 24 months	−0.04	(−0.14, 0.07)	−0.03	(−0.14, 0.07)	−0.07	(−0.17, 0.03)
3 to 36 months	−0.04	(−0.13, 0.06)	−0.04	(−0.13, 0.06)	−0.07	(−0.17, 0.03)
WLZ						
3 to 12 months	−0.02	(−0.15, 0.12)	−0.01	(−0.15, 0.13)	−0.04	(−0.18, 0.09)
3 to 24 months	−0.03	(−0.13, 0.08)	−0.02	(−0.12, 0.09)	−0.06	(−0.16, 0.05)
3 to 36 months	−0.02	(−0.12, 0.08)	−0.01	(−0.11, 0.09)	−0.05	(−0.16, 0.05)

Abbreviation: β, regression coefficient; CI, confidence interval; LAZ, length for age Z scores; WAZ, weight for age Z scores; WLZ, weight for length Z scores. Generalized estimating equation models were used to assess the effects. ^a^ Model 1 included the variables of maternal age, maternal education, average monthly household income, and infant gender. ^b^ Model 2 included the variables of Model 1 plus maternal postpartum BMI, maternal height, maternal calcium supplementation, gestational dietary intake, passive smoking during pregnancy, alcohol use during pregnancy, maternal parity, infant birthweight, length at birth, gestational age, infant feeding practices, infant supplement use, and infant dietary intake.

**Table 4:** Effects of maternal prenatal multi-micronutrient supplementation compared with iron–folic acid supplementation on nutritional status at different age points.

Outcomes	At Birth	3 Months	6 Months	12 Months	18 Months	24 Months	36 Months
Underweight							
Unadjusted	0.34(0.04, 3.04)	3.87(0.41, 36.97)	3.77(0.89, 6.53)	1.56(0.82, 2.96)	3.01(0.56, 16.29)	1.63(0.84, 3.16)	1.47(0.75, 2.86)
Model 1 ^a^	0.31(0.04, 2.80)	3.84(0.40, 36.87)	3.69(0.85, 6.37)	1.53(0.80, 2.92)	3.02(0.56, 16.44)	1.55(0.80, 3.01)	1.61(0.82, 3.15)
Model 2 ^b^	0.22(0.02, 2.24)	4.72(0.49, 45.74)	3.52(0.74, 6.04)	1.43(0.75, 2.72)	3.83(0.63, 23.24)	1.46(0.75, 2.85)	1.48(0.73, 2.92)
Stunting							
Unadjusted	0.68(0.13, 3.70)	1.29(0.33, 5.11)	2.61(0.24, 28.64)	1.29(0.18, 9.12)	0.60(0.12, 3.07)	2.87(0.73, 11.34)	1.41(0.09, 22.39)
Model 1 ^a^	0.69(0.13, 3.79)	1.28(0.32, 5.05)	2.52(0.23, 27.36)	1.07(0.15, 7.45)	0.65(0.13, 3.23)	3.11(0.79, 12.35)	1.30(0.08, 20.58)
Model 2 ^b^	1.12(0.15, 8.25)	1.36(0.34, 5.49)	3.30(0.27, 41.13)	1.22(0.13, 11.34)	0.54(0.09, 3.26)	5.55(1.19, 26.03)	1.35(0.09, 19.82)
Wasting							
Unadjusted	1.05(0.47, 2.36)	1.27(0.26, 6.25)	1.43(0.67, 3.09)	1.68(0.71, 4.01)	1.51(0.38, 5.95)	2.15(0.36, 12.76)	0.47(0.05, 4.48)
Model 1 ^a^	1.02(0.46, 2.29)	1.29(0.26, 6.37)	1.49(0.69, 3.22)	1.54(0.64, 3.67)	1.53(0.39, 6.04)	1.70(0.28, 10.40)	0.36(0.04, 3.58)
Model 2 ^b^	0.89(0.38, 2.07)	1.12(0.23, 5.60)	1.35(0.64, 2.96)	1.34(0.56, 3.22)	1.92(0.44, 8.38)	2.77(0.25, 31.08)	0.53(0.06, 5.03)
Overweight							
Unadjusted	0.57(0.20, 1.59)	0.50(0.31, 0.81) **	0.85(0.53, 1.35)	0.92(0.49, 1.76)	1.20(0.58, 2.52)	1.18(0.50, 2.79)	1.22(0.60, 2.51)
Model 1 ^a^	0.58(0.20, 1.62)	0.51(0.32, 0.81) **	0.87(0.55, 1.39)	1.00(0.53, 1.89)	1.24(0.59, 2.59)	1.21(0.52, 2.86)	1.17(0.57, 2.41)
Model 2 ^b^	0.68(0.23, 2.00)	0.52(0.32, 0.84) **	0.93(0.57, 1.50)	0.89(0.44, 1.78)	1.40(0.65, 3.04)	1.04(0.41, 2.64)	1.16(0.54, 2.48)
Obesity							
Unadjusted	0.29(0.11, 0.74) *	0.54(0.29, 1.01)	0.92(0.56, 1.51)	0.97(0.51, 1.86)	1.15(0.57, 2.32)	0.61(0.24, 1.57)	1.76(0.84, 3.68)
Model 1 ^a^	0.28(0.11, 0.73) **	0.54(0.29, 1.01)	0.95(0.58, 1.56)	1.02(0.53, 1.94)	1.18(0.59, 2.38)	0.61(0.24, 1.57)	1.70(0.81, 3.56)
Model 2 ^b^	0.30(0.11, 0.78) *	0.54(0.29, 1.02)	0.99(0.59, 1.65)	1.09(0.54, 2.17)	1.18(0.58, 2.42)	0.55 (0.20, 1.53)	1.36(0.64, 2.91)

General linear models were used to estimate the effects. Values are the relative risk and 95% confidence intervals. Level of significance: * *p* < 0.05, ** *p* < 0.01. ^a^ Model 1 included the variables of maternal age, maternal education, average monthly household income, and infant gender. ^b^ Model 2 included the variables of Model 1 plus maternal postpartum BMI, maternal height, maternal calcium supplementation, gestational dietary intake, passive smoking during pregnancy, alcohol use during pregnancy, maternal parity, infant birthweight, length at birth, gestational age, infant feeding practices, infant supplement use, and infant dietary intake. At the age of birth, infant birthweight and length at birth were not adjusted.

**Table 5:** Effects of maternal prenatal multi-micronutrient supplementation compared with iron and folic acid supplementation on infant linear growth within different age intervals.

Outcomes	Unadjusted Analysis		Model 1 ^a^		Model 2 ^b^	
	RR	95% CI	RR	95% CI	RR	95% CI
Underweight						
3 to 12 months	3.90	(0.41, 37.13)	3.89	(0.41, 36.90)	3.85	(0.40, 37.19)
3 to 24 months	2.39	(0.71, 8.07)	2.41	(0.72, 8.11)	2.47	(0.63, 9.70)
3 to 36 months	1.93	(0.62, 6.01)	1.97	(0.65, 6.08)	1.86	(0.53, 6.56)
Stunting						
3 to 12 months	1.48	(0.51, 4.25)	1.47	(0.51, 4.24)	1.26	(0.41, 3.83)
3 to 24 months	1.41	(0.66, 3.01)	1.40	(0.66, 2.97)	1.32	(0.68, 2.58)
3 to 36 months	1.48	(0.69, 3.15)	1.48	(0.70, 3.14)	1.39	(0.64, 3.05)
Wasting						
3 to 12 months	0.92	(0.35, 2.37)	0.99	(0.37, 2.60)	0.85	(0.32, 2.37)
3 to 24 months	0.90	(0.34, 2.36)	0.95	(0.36, 2.51)	0.75	(0.26, 2.19)
3 to 36 months	0.83	(0.34, 2.02)	0.85	(0.35, 2.07)	0.73	(0.29, 1.85)
Overweight						
3 to 12 months	0.69	(0.47, 1.00)	0.71	(0.49, 1.03)	0.62	(0.43, 0.89) *
3 to 24 months	0.76	(0.54, 1.07)	0.79	(0.56, 1.12)	0.77	(0.55, 1.08)
3 to 36 months	0.78	(0.55, 1.10)	0.80	(0.57, 1.13)	0.72	(0.51, 1.02)
Obesity						
3 to 12 months	0.77	(0.51, 1.16)	0.80	(0.54, 1.20)	0.78	(0.53, 1.15)
3 to 24 months	0.78	(0.54, 1.11)	0.82	(0.58, 1.17)	0.78	(0.55, 1.11)
3 to 36 months	0.87	(0.63, 1.21)	0.91	(0.66, 1.26)	0.82	(0.60, 1.12)

Generalized estimating equation models were used to estimate the effects. Values are the relative risk and 95% confidence intervals. Level of significance: * *p* < 0.05. ^a^ Model 1 included the variables of maternal age, maternal education, average monthly household income, and infant gender. ^b^ Model 2 included the variables of Model 1 plus maternal postpartum BMI, maternal height, maternal calcium supplementation, gestational dietary intake, passive smoking during pregnancy, alcohol use during pregnancy, maternal parity, infant birthweight, length at birth, gestational age, infant feeding practices, infant supplement use, and infant dietary intake.

**Table 6:** Effects of maternal prenatal multi-micronutrient supplementation compared with iron–folic acid supplementation on development delay at 36 months old.

Outcomes	Unadjusted Analysis		Model 1 ^a^		Model 2 ^b^	
	RR	95% CI	RR	95% CI	RR	95% CI
Communication	1.24	(0.47, 3.25)	1.16	(0.44, 3.03)	0.98	(0.34, 2.81)
Gross motor	0.52	(0.19, 1.44)	0.48	(0.17, 1.33)	0.48	(0.17, 1.38)
Fine motor	0.58	(0.26, 1.33)	0.62	(0.27, 1.40)	0.52	(0.22, 1.21)
Problem solving	0.93	(0.49, 1.77)	0.93	(0.49, 1.78)	1.06	(0.53, 2.10)
Personal social	0.75	(0.47, 1.19)	0.73	(0.46, 1.16)	0.66	(0.41, 1.09)
Total development	0.80	(0.56, 1.12)	0.78	(0.56, 1.10)	0.77	(0.54, 1.11)

General linear models were used to assess the effects. ^a^ Model 1 included the variables of maternal age, maternal education, average monthly household income, and infant gender. ^b^ Model 2 included the variables of Model 1 plus maternal postpartum BMI, maternal height, maternal calcium supplementation, gestational dietary intake, passive smoking during pregnancy, alcohol use during pregnancy, maternal parity, infant birthweight, length at birth, gestational age, infant feeding practices, infant supplement use, and infant dietary intake.

## Supplementary Materials

The following are available online at https://www.mdpi.com/1660-4601/16/15/2744/s1, Table S1: Maternal and child characteristics with missing values according to prenatal micronutrient supplementation status, Table S2: The mean LAZs, WAZs, and WLZs by infant age in the cohort, Table S3: Children’s development score and delay at age of 3 years in the cohort.

## References

[1] Haider B.A., Bhutta Z.A. (2017). Multiple-micronutrient supplementation for women during pregnancy. Cochrane Database Syst. Rev..

[2] World Health Organization (2016). Guideline: Use of Multiple Micronutrient Powders for Home Fortification of Foods Consumed by Pregnant Women.

[3] Christian P., Stewart C.P. (2010). Maternal micronutrient deficiency, fetal development, and the risk of chronic disease. J. Nutr..

[4] World Health Organization (2016). Guideline: WHO Recommendations on Antenatal Care for a Positive Pregnancy Experience.

[5] World Health Organization, United Nations Children’s Fund (2007). Reaching Optimal Iodine Nutrition in Pregnant and Lactating Women and Young Children: A Joint Statement by the World Health Organization and the United Nations Children’s Fund.

[6] Yu X., Chen W., Wei Z., Ren T., Yang X., Yu X. (2016). Effects of maternal mild zinc deficiency and different ways of zinc supplementation for offspring on learning and memory. Food Nutr. Res..

[7] Palmer A.C., Schulze K.J., Khatry S.K., De Luca L.M., West K.P.J. (2015). Maternal vitamin A supplementation increases natural antibody concentrations of preadolescent offspring in rural Nepal. Nutrition.

[8] Zerfu T.A., Ayele H.T. (2013). Micronutrients and pregnancy; effect of supplementation on pregnancy and pregnancy outcomes: A systematic review. Nutr. J..

[9] West K.P.J., Shamim A.A., Mehra S., Labrique A.B., Ali H., Shaikh S., Klemm R.D., Wu L.S., Mitra M., Haque R. (2014). Effect of maternal multiple micronutrient vs. iron-folic acid supplementation on infant mortality and adverse birth outcomes in rural Bangladesh: The JiVitA-3 randomized trial. JAMA.

[10] Devakumar D., Fall C.H., Sachdev H.S., Margetts B.M., Osmond C., Wells J.C., Costello A., Osrin D. (2016). Maternal antenatal multiple micronutrient supplementation for long-term health benefits in children: A systematic review and meta-analysis. BMC Med..

[11] Dennison E., Fall C., Cooper C., Barker D. (1997). Prenatal factors influencing long-term outcome. Horm. Res..

[12] Barker D.J., Gluckman P.D., Godfrey K.M., Harding J.E., Owens J.A., Robinson J.S. (1993). Fetal nutrition and cardiovascular disease in adult life. Lancet.

[13] Black M.M., Walker S.P., Fernald L.C.H., Andersen C.T., DiGirolamo A.M., Lu C., McCoy D.C., Fink G., Shawar Y.R., Shiffman J. (2017). Lancet Early Childhood Development Series Steering Committee. Early childhood development coming of age: Science through the life course. Lancet.

[14] Christian P., Kim J., Mehra S., Shaikh S., Ali H., Shamim A.A., Wu L., Klemm R., Labrique A.B., West K.P.J. (2016). Effects of prenatal multiple micronutrient supplementation on growth and cognition through 2 y of age in rural Bangladesh: The JiVitA-3 Trial. Am. J. Clin. Nutr..

[15] Huy N.D., Le Hop T., Shrimpton R., Hoa C.V. (2009). An effectiveness trial of multiple micronutrient supplementation during pregnancy in Vietnam: Impact on birthweight and on stunting in children at around 2 years of age. Food Nutr. Bull..

[16] Vaidya A., Saville N., Shrestha B.P., Costello A.M., Manandhar D.S., Osrin D. (2008). Effects of antenatal multiple micronutrient supplementation on children’s weight and size at 2 years of age in Nepal: Follow-up of a double-blind randomised controlled trial. Lancet.

[17] Roberfroid D., Huybregts L., Lanou H., Ouedraogo L., Henry M.C., Meda N., Kolsteren P. (2012). MISAME study group. Impact of prenatal multiple micronutrients on survival and growth during infancy: A randomized controlled trial. Am. J. Clin. Nutr..

[18] Wang W., Yan H., Zeng L., Cheng Y., Wang D., Li Q. (2012). No effect of maternal micronutrient supplementation on early childhood growth in rural western China: 30 month follow-up evaluation of a double blind, cluster randomized controlled trial. Eur. J. Clin. Nutr..

[19] Li Q., Yan H., Zeng L., Cheng Y., Liang W., Dang S., Wang Q., Tsuji I. (2009). Effects of maternal multimicronutrient supplementation on the mental development of infants in rural western China: Follow-up evaluation of a double-blind, randomized, controlled trial. Pediatrics.

[20] Sha T., Gao X., Chen C., Li L., He Q., Wu X., Cheng G., Tian Q., Yang F., Yan Y. (2019). Associations of Pre-Pregnancy BMI, Gestational Weight Gain and Maternal Parity with the Trajectory of Weight in Early Childhood: A Prospective Cohort Study. Int. J. Environ. Res. Public Health.

[21] Wu X., Gao X., Sha T., Zeng G., Liu S., Li L., Chen C., Yan Y. (2019). Modifiable Individual Factors Associated with Breastfeeding: A Cohort Study in China. Int. J. Environ. Res. Public Health.

[22] Schlesselman J.J. (1974). Sample size requirements in cohort and case-control studies of disease. Am. J. Epidemiol..

[23] World Health Organization (2006). WHO Child Growth Standards: Length/Height-Forage, Weight-for-Age, Weight-for-Length, Weight-for-Height and Body Mass Index-for-Age: Methods and Development.

[24] Squires J., Bricker D. (2009). Ages & Stages Questionnaires (ASQ-3).

[25] Wei M., Bian X., Squires J., Yao G., Wang X., Xie H., Song W., Lu J., Zhu C., Yue H. (2015). Studies of the norm and psychometrical properties of the ages and stages questionnaires, third edition, with a Chinese national sample. Zhonghua Er Ke Za Zhi.

[26] Xie H., Clifford J., Squires J., Chen C.Y., Bian X., Yu Q. (2017). Adapting and validating a developmental assessment for chinese infants and toddlers: The ages & stages questionnaires: Inventory. Infant Behav. Dev..

[27] Zhou B.F. (2002). Cooperative Meta-Analysis Group of the Working Group on Obesity in China. Predictive values of body mass index and waist circumference for risk factors of certain related diseases in Chinese adults–study on optimal cut-off points of body mass index and waist circumference in Chinese adults. Biomed. Environ. Sci..

[28] Lange S., Probst C., Quere M., Rehm J., Popova S. (2015). Alcohol use, smoking and their co-occurrence during pregnancy among Canadian women, 2003 to 2011/12. Addict. Behav..

[29] Lee K.J., Carlin J.B. (2010). Multiple imputation for missing data: Fully conditional specification versus multivariate normal imputation. Am. J. Epidemiol..

[30] Spratt M., Carpenter J., Sterne J.A., Carlin J.B., Heron J., Henderson J., Tilling K. (2010). Strategies for multiple imputation in longitudinal studies. Am. J. Epidemiol..

[31] Yang J., Cheng Y., Pei L., Jiang Y., Lei F., Zeng L., Wang Q., Li Q., Kang Y., Shen Y. (2017). Maternal iron intake during pregnancy and birth outcomes: A cross-sectional study in Northwest China. Br. J. Nutr..

[32] United Nations International Children’s Emergency Fund, World Health Organization, United Nations Children’s Fund (1999). Composition of a Multi-Micronutrient Supplement to be Used in Pilot Programmes Among Pregnant Women in Developing Countries.

[33] Prado E.L., Sebayang S.K., Apriatni M., Adawiyah S.R., Hidayati N., Islamiyah A., Siddiq S., Harefa B., Lum J., Alcock K.J. (2017). Maternal multiple micronutrient supplementation and other biomedical and socioenvironmental influences on children’s cognition at age 9–12 years in Indonesia: Follow-up of the SUMMIT randomised trial. Lancet Glob. Health.

[34] Zeng L., Dibley M.J., Cheng Y., Dang S., Chang S., Kong L., Yan H. (2008). Impact of micronutrient supplementation during pregnancy on birth weight, duration of gestation, and perinatal mortality in rural western China: Double blind cluster randomised controlled trial. BMJ.

[35] Kaestel P., Michaelsen K.F., Aaby P., Friis H. (2005). Effects of prenatal multimicronutrient supplements on birth weight and perinatal mortality: A randomised, controlled trial in Guinea-Bissau. Eur. J. Clin. Nutr..

[36] Fall C.H., Fisher D.J., Osmond C., Margetts B.M. (2009). Multiple micronutrient supplementation during pregnancy in low-income countries: A meta-analysis of effects on birth size and length of gestation. Food Nutr. Bull..

[37] Tanner J.M. (1981). Catch-up growth in man. Br. Med. Bull..

[38] Bhargava A. (2016). Protein and Micronutrient Intakes Are Associated with Child Growth and Morbidity from Infancy to Adulthood in the Philippines. J. Nutr..

[39] Adamo A.M., Oteiza P.I. (2010). Zinc deficiency and neurodevelopment: The case of neurons. Biofactors.

[40] Skeaff S.A. (2011). Iodine Deficiency in Pregnancy: The Effect on Neurodevelopment in the Child. Nutrients.

[41] McGrath N., Bellinger D., Robins J., Msamanga G.I., Tronick E., Fawzi W.W. (2006). Effect of maternal multivitamin supplementation on the mental and psychomotor development of children who are born to HIV-1–infected mothers in Tanzania. Pediatrics.

[42] Sanstead H.H. (1985). W.O. Atwater memorial lecture. Zinc: Essentiality for brain development and function. Nutr. Rev..

[43] Dong J., Yin H., Liu W., Wang P., Jiang Y., Chen J. (2005). Congenital iodine deficiency and hypothyroidism impair LTP and decrease C-fos and C-jun expression in rat hippocampus. Neurotoxicology.

[44] Navarro D., Alvarado M., Navarrete F., GIner M., Obregon M.J., Manzanares I., Berbel P. (2015). Gestational and early postnatal hypothyroidism alters VGLuT1 and VGAT bouton distribution in the neocortex and hippocampus, and behavior in rats. Front. Neuroanat..

[45] Guilarte T.R., Wagner H.N., Frost J.J. (1987). Effects of perinatal vitamin B6 deficiency on dopaminergic neurochemistry. J. Neurochem..

[46] Potdar R.D., Sahariah S.A., Gandhi M., Kehoe S.H., Brown N., Sane H., Dayama M., Jha S., Lawande A., Coakley P.J. (2014). Improving women’s diet quality preconceptionally and during gestation: Effects on birth weight and prevalence of low birth weight–a randomized controlled efficacy trial in India (Mumbai maternal nutrition project). Am. J. Clin. Nutr..

