# Supplementary material for: Effects of Maternal Prenatal Multi-Micronutrient Supplementation on Growth and Development until 3 Years of Age
Source: Int J Environ Res Public Health. 2019 Aug 1;16(15):2744. doi: 10.3390/ijerph16152744 (PMC6696317; doi:10.3390/ijerph16152744)
Supplement: Supplementary file 1 [file ijerph-16-02744-s001.pdf]

Table S1. Maternal and child characteristics with missing values according to prenatal micronutrient supplementation status.

Characteristics	MM (*n* = 250)	IFA (*n* = 343)	FA (*n* = 339)	*p*
Maternal characteristics				
Age (years)				0.603
<25	9(4.0)	14(3.8)	22(6.5)	
25-29	127(48.4)	158(49.7)	157(46.6)	
30-34	84(35.4)	123(31.6)	115(34.4)	
≥35	30(12.2)	47(14.6)	43(12.8)	
Education				0.005 *
Junior school or below	5(2.0)	12(3.6)	15(4.5)	
Senior high school	22(8.8)	36(10.7)	58(17.5)	
College or above	222(89.2)	289(85.8)	259(78.0)	
Average monthly household income (RMB)				0.113
≤2000	10(4.2)	10(3.0)	11(3.3)	
2001-5000	125(52.5)	160(48.2)	193(58.0)	
>5000	103(43.3)	162(48.8)	129(38.7)	
Postpartum BMI (kg/m^2)				0.735
<18.5	5(2.1)	6(1.8)	5(1.5)	
18.5-23.9	134(56.8)	196(60.3)	182(55.7)	
24-27.9	77(32.6)	101(31.1)	106(32.4)	
≥28	20(8.5)	22(6.8)	34(10.4)	
Height (m)	1.60 ± 0.08	1.60 ± 0.07	1.60 ± 0.04	0.670
Parity				0.013 *
Primiparous	192(76.8)	224(65.7)	233(69.1)	
Multiparous	58(23.2)	117(34.3)	107(30.9)	
Infant characteristics				
Gender				0.327
Male	138(55.2)	177(51.6)	166(49.0)	
Female	112(44.8)	166(48.4)	173(51.0)	
Birthweight (g)				0.814
<2500	3(1.2)	5(1.5)	8(2.4)	
2500-3999	227(91.5)	311(92.3)	307(91.1)	
≥4000	18(7.3)	21(6.2)	22(6.5)	
Length at birth (cm)	50.00 ± 1.01	49.98 ± 0.78	49.88 ± 0.92	0.201
Gestational age (weeks)				0.923
<37	9(3.7)	10(3.0)	13(3.9)	
37-41	236(95.9)	319(96.4)	321(95.3)	
≥42	1(0.4)	2(0.6)	3(0.9)	

Abbreviation: MM, multi-micronutrient; IFA, iron-folic acid; FA, folic acid. Values are *n* (%) or means ± SDs. One-way ANOVA was used to compare means; the chi-square test was used to compare proportions. Level of significance: $^*p < 0.05$.

Table S2. The mean LAZs, WAZs, and WLZs by infant age in the cohort.

Outcomes	Total	At Birth	3 Months	6 Months	12 Months	18 Months	24 Months	36 Months
LAZ	0.30 ± 1.01	0.24 ± 0.52	0.54 ± 1.11	0.46 ± 1.03	0.26 ± 1.02	0.17 ± 1.01	0.17 ± 0.95	0.29 ± 0.91
WAZ	0.44 ± 0.99	0.22 ± 0.92	0.76 ± 1.08	0.70 ± 1.04	0.47 ± 0.91	0.31 ± 1.26	0.27 ± 0.87	0.30 ± 0.95
WLZ	0.34 ± 1.17	0.04 ± 1.36	0.51 ± 1.10	0.62 ± 1.06	0.42 ± 0.98	0.26 ± 1.50	0.22 ± 0.91	0.30 ± 1.09
Underweight	69(1.2)	8(0.9)	8(0.9)	9(1.0)	10(1.1)	11(1.3)	12(1.4)	11(1.6)
Stunting	70(1.2)	8(0.9)	14(1.6)	10(1.1)	5(0.6)	14(1.6)	15(1.8)	4(0.6)
Wasting	79(1.3)	40(4.3)	4(0.4)	5(0.5)	4(0.5)	15(1.8)	5(0.6)	6(0.9)
Overweight	373(6.2)	25(2.7)	99(11.1)	91(10.1)	50(5.7)	42(4.9)	27(3.2)	39(5.5)
Obesity	359(6.0)	47(5.0)	63(7.0)	85(9.4)	48(5.5)	46(5.4)	28(3.3)	42(6.0)

Abbreviation: LAZ: length for age Z scores, WAZ: weight for age Z scores, WLZ: weight for length Z scores. Values are means ± SDs or *n* (%).

Table S3. Children's development score and delay at age of 3 years in the cohort.

Outcomes	Means ± SD or *n* (%)
Communication score	58.46 ± 4.88
Gross motor score	58.62 ± 4.12
Fine motor score	52.32 ± 9.70
Problem solving score	55.32 ± 7.26
Personal social score	51.58 ± 7.81
Communication delay	21(3.0)
Gross motor delay	22(3.2)
Fine motor delay	32(4.6)
Problem solving delay	50(7.2)
Personal social delay	91(13.1)
Total development delay	150(21.6)

Values are Means ± SD or *n* (%).
